# Mathematics self-concept and challenges of learners in an online learning environment during COVID-19 pandemic

**DOI:** 10.1186/s40561-021-00168-5

**Published:** 2021-10-16

**Authors:** Rex Bringula, Jon Jester Reguyal, Don Dominic Tan, Saida Ulfa

**Affiliations:** 1 University of the East, CM Recto Avenue, 2219 Sampaloc, Manila, Philippines; 2 Educational Technology Department, Faculty of Education, Universitas Negeri Malang, Malang, Indonesia

**Keywords:** Ability, Learning management system, Mathematics, Physical learning space

## Abstract

In this mixed-methods research, the relationship between four factors of individual online learners and their mathematics self-concept was explored. In addition, the challenges the students faced in learning mathematics online during the Coronavirus disease (COVID-19) pandemic were determined. The participant students were from two mathematics classes offered online during the summer of 2020. Pure online classes were first offered during this period because face-to-face learning sessions were suspended due to the COVID-19 pandemic. It was found that students owned the devices they were using for online classes. Internet connection and power interruption were the most problematic aspects of online learning. Students had positive as well as negative mathematics online learning self-concepts. Individual factors were partly related to mathematics self-concept. Qualitative data shows that students faced technological, personal, domestic, assessment, pedagogical, consultation, and test anxiety challenges. Implications and recommendations for teaching mathematics in an online environment are offered.

## Introduction

Academic self-concept (ASC) is one's academic self-perceptions of one's general ability in school (Shavelson et al., 1976). It also refers to the interests of the students towards a particular course (Joyce & Yates, 2007). This idea has been widely investigated in the field of mathematics that eventually forms a field of study named mathematics self-concept (Lee & Kung, 2018; Pajares & Miller, 1994; Reyes, 1984). Mathematics self-concept has “to do with how sure a person is of being able to learn new topics in mathematics, perform well in mathematics class, and do well on mathematics tests” (p. 560, Reyes, 1984). It also refers to an individual's perception of his/her abilities related to mathematics as compared to others (Bong & Skaalvik, 2003). It is related to mathematics achievement (Lee & Kung, 2018). Thus, it is important to understand how students perceived their mathematics learning abilities.

However, these studies (e.g., Bong & Skaalvik, 2003; Lee & Kung, 2018; Pajares & Miller, 1994; Reyes, 1984) were investigated in the context of face-to-face learning where students can seek immediate learning interventions from teachers or their classmates. Moreover, while prior works (e.g., Baticulon et al., 2021; Fabito et al., 2021) outlined the barriers to online learning in the context of the COVID-19 pandemic, the mathematics self-concept in the context of online learning is not yet investigated. The sudden shift from face-to-face to online learning platforms posed challenges to students. Shifting to an online learning platform requires access to a device, Internet, and physical learning space, and a strong habit of learner’s autonomy. Due to the digital divide, device ownership is a persistent challenge in the effective implementation of online learning. For developing countries, intermittent Internet connection remains a barrier to effective online learning (Salac & Kim, 2016).

The shift from a classroom environment to a home learning environment raises another concern for students. Students that have no access to a personal physical online learning environment could be disrupted by noise and other distractions (Baticulon et al., 2021; Bringula et al., 2021). The pedagogical style also changed. In particular, there are parts of the content of the syllabus that students have to learn on their own (i.e., asynchronous session)—tasks that may not be a practice in a face-to-face setup. The ability of the students to learn and study course material during asynchronous sessions poses difficulties to online learners. These barriers are found to hinder the effective implementation of online learning. However, it is unclear whether these variables have a significant relationship with the students’ mathematics online learning self-concept (i.e., perceived abilities of the students to learn online mathematics online courses).

In other words, while technological, personal, institutional, and community barriers are identified in online learning in this time of the COVID-19 pandemic (Baticulon et al., 2021; Fabito et al., 2021), the relationship of these constraints to the mathematics self-concept of the students in a fully online class is still unknown. In addition, mathematics self-concept in an online learning setup is not considered in the existing guidelines on improving mathematics online education (e.g., Lee & Kung, 2018). Understanding the mathematics self-concept of the students at the early stages of the implementation of online learning could inform educators to improve or diversify their mathematics online teaching strategies. Hence, it is imperative to understand the relationship of these variables with the mathematics self-concept of the students in an online learning environment.

The findings of this study will benefit the teachers, the students, and the schools. Teachers may apply the recommended approach derived from the findings of the study in improving their online pedagogies. Schools may utilize the findings of the study in providing institutionalized strategies in addressing students’ online learning mathematics self-concept. In turn, students' online mathematics self-concept may be improved and students' mathematics learning may be achieved. Consequently, students' attrition to mathematics online learning courses may be lessened.

This study attempted to contribute to the existing threads of discussion of mathematics self-concept in the context of a fully online learning environment. The study investigated the mathematics self-concept of two classes of computing students during the summer of 2020. The results of the study served as a basis in the formulation of a checklist of suggestions for online mathematics teachers. Specifically, the study aims to answer the following research questions (RQ). (RQ) (1) What are the online learners-related factors in terms of device ownership, perceived Internet speed, personal physical learning space access, and mathematics learning autonomy? (2) What is the mathematics self-concept of online learners in terms of mathematics ability, mathematics interest, and perceived mathematics performance? (3) Is there a significant relationship between learner-related factors and mathematics self-concept? (4) Is there a significant difference in the mathematics self-concept of online learners between those with personal learning space and those without personal learning space?, and (5) What are the experiences of the respondents in terms of challenges in online learning?

## Literature review

### Online mathematics education

Berge et al. (2000) set forth the following recommendations for teachers to ensure effective implementation of online learning courses: (1) state the hardware and software requirements of the course, (2) be available for consultation, (3) be creative in interacting with students online, (4) provide course performance feedback, (5) listen to student concerns and encourage class participation, (6) establish clear policies, goals, course objectives, and course expectations, (7) be knowledgeable with the online learning software, (8) use different pedagogical styles, (9) encourage collaboration among students, and (10) be proactive and solve problems to avoid escalation. Meanwhile, Herrington et al. (2004) suggested that online activities should be composed of relevant, ill-defined, varied, and complex tasks that could be integrated across different subject areas. Moreover, teachers may also provide activities supporting opportunities for collaboration and reflection.

Different studies reported the factors that influenced the achievement of online mathematics education students. The results of these studies served as focal points on improving online mathematics teaching. The study of Wadsworth et al. (2007) disclosed that four learning strategies (motivation, concentration, information processing, and self-testing) and self-efficacy predicted online mathematics grade achievement. They suggested that online mathematics educators provided real-world examples and conduct meetings with students regarding learning strategies. In a similar study, Glass and Sue (2008) showed that assignments were the most preferred learning object and had the most impact on learning. Thus, adequate practice drills and timely feedback were necessary for online mathematics education. The findings of Wadsworth et al. (2007) and Glass and Sue (2008) are consistent with the guidelines of Herrington et al. (2004).

Güzeller and Akin (2012) compared the mathematics achievement, attitudes, anxiety, and self-efficacy of students in the web-based mathematics instructions (WBMI) and traditional mathematics instructions (TMI). It was found that there was a significant difference between the WBMI and TMI in terms of achievement, attitudes, anxiety, and self-efficacy, having more favorable results on the WBMI. Students were advised to take the WBMI to familiarize themselves with this platform. Kim et al. (2014) extended the study of Güzeller and Akin (2012) by investigating the impact of anxiety and other forms of academic emotions (anger, shame, boredom, enjoyment, and pride) on online mathematics achievement. Kim et al. (2014) further hypothesized that motivation, self-efficacy, and cognitive processes (e.g., cognitive strategy, self-regulated learning) influenced online mathematics achievement. Academic emotions were accounted for 37% of the variation in student achievement.

Finally, Cho and Heron (2015) determined the impact of self-regulated learning (SRL), learning strategies, and emotions on satisfaction with online learning and online mathematics achievement. The study revealed that motivation influenced achievement, and both motivation and emotion were related to satisfaction. Based on these findings, the study formulated the following recommendations for teachers: enhance students' self-efficacy, design supporting tools in online courseware, provide course orientation, provide SRL support through social media, and restructure the format of the course.

### Academic self-concept, academic self-efficacy, and mathematics self-concept

Arens et al. (2020) discussed the similarity and the difference between academic self-concept and academic self-efficacy. In general, both academic self-concept and academic self-efficacy address students' competence (Bong & Skaalvik, 2003 cited in Arens et al., 2020). However, academic self-concept is related to the self-perceived competence of a student in an academic domain in general (e.g., math; Marsh & Craven, 2006). Meanwhile, academic self-efficacy is self-perceived confidence to perform successfully a given task in a specific domain (e.g., Bandura, 2001; Zimmerman, 2000). In other words, self-concept is a domain-specific construct while self-efficacy is a domain- and task-specific construct (Arens et al., 2020).

Self-concept is investigated in the field of mathematics. Mathematics self-concept is attributed to students’ aspirations to pursue degree programs in STEM (science, technology, engineering, and mathematics) (Sax et al., 2015). Students with positive mathematics self-concept stay and finish their chosen degree program which, in turn, contributes to improving the school's student retention (Ackerman et al., 2013). This can be explained by the findings that mathematics self-concept is positively related to mathematics achievement (Kung, 2009; Lee & Kung, 2018). Moreover, mathematics self-concept and mathematics self-efficacy (i.e., a belief of student’s ability in solving mathematical problems or tasks related to mathematics; Masitoh & Firtriyani, 2018) predicted mathematics achievement (Kung, 2009). Thus, it is necessary to understand the mathematics self-concept of the students since it serves as a basis to cultivate the students’ learning interests in the program.

The measurement of mathematics self-concept is primarily based on the prior works of self-concept (e.g., Marsh & O'Neill, 1984; Marsh et al., 1983, 2005). The questionnaire was composed of different areas including mathematics, verbal abilities, academic capabilities, problem-solving/creativity, physical abilities/sports, physical appearance, relations with same-sex peers, relations with opposite-sex peers, relations with parents, religion/spirituality, honesty/reliability, emotional stability/security, and general self-concept. The mathematics factor was composed of items such as “I find many mathematical problems interesting and challenging”, “Mathematics makes me feel inadequate”, “I am quite good at mathematics”, “I have trouble understanding anything that is based upon mathematics”, and “I have always done well in mathematics classes”. Recent studies on mathematics self-concept based their works on self-concept questionnaire developed by Marsh and colleagues (Marsh & O'Neill, 1984; Marsh et al., 1983, 2005). For example, Lee and Kung (2018) investigated the mathematics self-concept and mathematics achievement of junior high school students in Taiwan. The authors devised a 13-item questionnaire measuring competence in mathematics, affection towards mathematics, and comparison of mathematics abilities.

### Barriers to online learning

Cavanaugh et al. (2009) reported the barriers in the existing literature to online learning implementation as well as the benefits and challenges of online learning. Higher levels of motivation, expanding educational access, providing high-quality learning opportunities, improving student outcomes and skills, allowing educational choice, and administrative efficiency are notable benefits of online learning. Meanwhile, the high cost of start-ups, digital divide issues, governmental approval, and student readiness are the challenges raised in the implementation of online learning.

In a recent study, Binti Abd Aziz et al. (2020) investigated the barriers to online learning. According to the authors, addressing these barriers could lead to effective online learning practices. They identified the barriers in terms of attitudes, interruptions, technology skills, and personal skills. Attitudes towards online learning refer to the feelings of the people towards online learning. Computer, online, and computer application literacy skills are the components of technology skills. Interruptions to online learning are defined as the limits to technological access because students may be living in rural areas, being part of a minority group, having disabilities, or due to mature age. Personal skills are skills relating to prior experience of using online learning. Path analysis disclosed that attitudes toward online learning and technology skills are the main barriers to online learning.

The inability of the students to study at their own pace has also posed a barrier to students' online learning. Learner autonomy is the ability of learners to assume control or to take charge of their learning (Benson, 2001). Autonomous learners were able to understand the online learning process (Fotiadou et al., 2017), which enabled them to achieve high grades in online learning classes (Yen & Liu, 2009). In other words, mathematics learner autonomy is the ability of learners to understand and assume control learning of the online materials with little supervision (Benson, 2001; Fotiadou et al., 2017).

In the Philippines, Pena-Bandalaria (2009) reported that personal concerns (e.g., difficulty to interact and contact teachers, difficulty to seek help, difficulty in understanding the topics), technical concerns (e.g., problems accessing the course site), and the digital divide were barriers to online learning. This finding is consistent with the results of the study of Gledhill et al. (2017). Gledhill et al. (2017) also revealed that limited or poor access to the Internet, technology, and networks were the constraints of e-learning in less developed countries. Perceived Internet speed is the subjective evaluation of the speed of the Internet in supporting online learning sessions (Gledhill et al., 2017). Natividad (2021), and Salac and Kim (2016) explained the slow Internet connection in the Philippines. They agreed that Internet connection in the Philippines is slow due to limited Internet infrastructure which is brought by outdated laws and heavy bureaucratic processes for the development of Internet infrastructure. Furthermore, intermittent power supply was a major problem that hinders e-learning implementation in less developed countries (Bhuasiri et al., 2012).

In this time of COVID-19 pandemic, Philippine higher education institutions also implemented emergency online learning programs (Murphy, 2020). The emergency implementation may caught students unprepared (Aguilera-Hermida, 2020; Daniel, 2020). Baticulon et al. (2021) reported the major barriers of Filipino medical students to adopt online learning. These barriers can be classified as technological (lack of devices, issues with the online platform, Internet connectivity), individual (students’ learning style, physical and mental health), domestic (concerns at home, financial distress), institutional (school curriculum), and community barriers (lockdown restrictions, infrastructure challenges, and sociopolitical issues). Students found it difficult to understand the learning materials on their own. It was also reported that students had difficulty studying at home because of noise, distractions, and small space. Personal physical learning space refers to the space dedicated to online learning that is free from distraction or noise (Baticulon et al., 2021).

In a similar study of Fabito et al. (2021), they found that difficulty of clarifying topics or discussions with the professors, lack of study or working area dedicated for online activities, and lack of good Internet connection were the top three barriers and challenges that the 300 computing students (Computer Science and Information Technology) experienced. The study concluded that students and teachers were both not prepared to undergo full online learning. In a similar study, Bringula et al. (2021) found that the number of owned devices had a positive influence on the perceived academic online learning performance of computing students. Device ownership refers to the number of devices students own in accessing the LMS (Bringula et al., 2021). It was shown that students that own multiple devices are more likely to have positive dispositions towards their academic online learning performance than those students who have difficulty access to online learning devices.

### Synthesis of literature review

There is a wealth of studies proposing the effective delivery of mathematics online education. These recommendations did not consider students' mathematic online self-concept. Moreover, the recommended teaching strategies are not set forth in the context of COVID-19 pandemic where students faced physical and psychological challenges (Baticulon et al., 2021; Bringula et al., 2021; Gledhill et al., 2017; Pena-Bandalaria, 2009). In particular, the continuous lockdowns in the Philippines exacerbate the existing phenomenon of digital divide, e.g., students cannot utilize public pay-for-access of computers and Internet in computer shops (Baticulon et al., 2021). Students may experience difficulty on engaging to mathematics online learning due to limited access to basic online resources. In turn, students may feel less capable of learning the mathematics online materials at their own pace. Determining the possible connection between barriers to online learning and students’ mathematics self-concept may help teachers, parents, and educational institutions to formulate pedagogical interventions to achieve desired online mathematics achievement.

## Methodology

### Research design, research setting, participants, sample, and data gathering procedure

This mixed-method study was conducted in one department of a university in Manila. In the quantitative part, the participants of the study were second-year college students (subsequently referred to as online learners) enrolled in two classes in Numerical Analysis. There were 69 online learners enrolled in the said course. This was the only mathematics course offered at the time the study was conducted. The study was conducted after the first week of full implementation of pure online learning through a learning management system (LMS). Learning materials and lecture sessions were both delivered in synchronous and asynchronous methods, but mostly delivered in asynchronous methods (36 h out of 54 h). Online learners were informed at the beginning of the online class sessions about this setup. The survey form was distributed in the LMS. An online survey form was distributed to all online learners but only 54 students participated in the study. Online learners in the study are composed of male (59%) and female (41%) students with an average age of 20 years old.

### Research instrument

The study utilized a content-validated survey form that served as a research instrument. The survey form consisted of two parts. The first part gathered information about the online learners profiles such as device ownership, perceived Internet speed, personal physical learning space ownership, and mathematics learning autonomy. They were also asked whether they have personal/private physical space for online learning (Baticulon et al., 2021). Perceived Internet speed can be answered using the responses “Very slow”, “Slow”, “Sometimes fast, sometimes slow”, “Fast”, and “Very fast”.

The mathematics learning autonomy intends to determine the level of independence to learn mathematics. It was measured using a 5-point scale in which the most negative response (i.e., total dependence to teachers or classmates) had an assigned value of 1 while the most positive response had a value of 5 (i.e., can independently learn the course content). These variables were selected because these were deemed relevant to the participants of the study and these were believed to influence engagement in online learning in the context of the COVID-19 pandemic (Pynos, 2016).

The second part solicited data on the mathematics self-concept of online learners. Mathematics self-concept consisted of mathematics ability (12 items), interest (2 items), and perceived mathematics performance (1 item). Online learners were asked about their perceived abilities and interest in mathematics learning when the course is delivered in an online setting. All items of ability and interest were preceded by the phrase “Considering that the course is delivered online,…”. The items could be answered using the responses “very untrue to me”, “untrue to me”, “unsure”, “true to me”, and “very true to me”. These verbal responses had assigned values from -2 to 2, where the most negative response has a value of -2 while the most positive response has a value of 2. Students were asked to complete the sentence “Considering that the course is delivered online, your mathematics grade will be…” to measure their perceived mathematics performance. This question could be answered using the responses “higher than face-to-face”, “about the same with face-to-face”, “lower than face-to-face”, and “not sure/cannot tell”.

The definition of academic self-concept relating to ability (Shavelson et al., 1976) and interest (Joyce & Yates, 2007) served as the basis in the construction of the research instrument. The items of the research instrument were adapted from Joyce and Yates (2007), Marsh et al. (1983), and Lee and Kung (2018). Only the ability component in the questionnaire of Lee and Kung (2018) was adopted in this study. The items applicable to online learning were retained. The adapted research instrument was then pilot-tested to 50 students who were not part of the study. Factor and Cronbach’s alpha analyses revealed that all items were found valid (factor loading ≥ 0.50) and reliable (Cronbach’s alpha α ≥ 0.70).

### Statistical treatment of data

Frequency counts, means, and cross-tabulation were used to describe the data. Spearman Rank correlation was employed to determine the relationship between the profile of the online learners and mathematics self-concept. Mann–Whitney U test was used to determine whether there is a significant difference between the mathematics self-concept of online learners when categorized by personal physical learning space access. A 0.05 level of significance was used to determine the significance of the results.

### Interview sessions, selection of interview participants, and participants

In the qualitative part, a series of separate interviews were conducted with three Information Technology (IT) students to further explain the findings of the study. The participants were selected based on their mathematics abilities and access to personal learning space. The authors sought the recommendations of the teachers who handled the course to identify the possible participants. The teachers identified and categorized the mathematics abilities of the students. These classifications are reliable because teachers know their students’ capabilities (Cheong et al., 2004; Lambert, 2002; Reeve, 2006). The participants consisted of 3 male, third-year students with an average age of 20 years old and they had varying degrees of mathematics abilities (i.e., struggling, average, and high performing). One of the informants (i.e., respondents) has no personal space dedicated to online learning. Two female participants were invited but they refused to participate in the study.

The interviews were conducted through Google Meet. Informants were interviewed one-by-one on different occasions to protect their identities. Students were asked about their challenges in online learning. The students were asked about their study practices in a face-to-face class (e.g., attending classes, review preparations for quizzes and exams, practicing solving math problems, reading materials, and taking lecture notes), challenges experienced in an online learning class, their perceived abilities in a face-to-face and online learning class, and their recommendations relating to the improvement of online pedagogy.

### Qualitative data analysis

The interviews were transcribed and were tabulated in a word processor. The tabulated results were then presented for validation. The purpose of the validation process was to determine whether other students had the same experiences. The validation of interview results was done through a presentation to another set of students (i.e., the validators) with the same level of mathematics abilities and also enrolled in the mathematics class during the summer period. Throughout these procedures, all identities of the participants were kept confidential. The validators were asked whether they agree or disagree with the responses collected from the interviews. The validators were composed of IT students (3 males with an average age of 20 years old; 2 third-year students and 1 s-year student; one of the respondents has no personal learning space). After the validation process, the tabulated responses were analyzed through qualitative content analysis (Hsieh & Shannon, 2005; Mayring, 2014).

The responses were coded and categorized based on Baticulon et al.'s (2021) classifications of barriers to online learning. The codes were keywords or phrases that represented the challenges of learning mathematics in an online environment or their recommendations to their teachers about teaching mathematics in an online setup. The authors also made their classifications if an item did not fit on the Baticulon et al.'s (2021) classification. The codes were then assigned to themes (i.e., challenges of online learning and the recommendations to improve online learning). One of the authors coded the responses. When coding the text, the coder was guided by the themes. The process was repeated until all keywords and phrases were assigned to the themes. Afterward, the research team deliberated whether they agree (or disagree) with the themes. In case of disagreement, the deliberation process was repeated until a consensus was reached (Bringula et al., 2019).

Both the quantitative and qualitative results of the study were presented during the department general faculty meeting to elicit feedback and inputs on how mathematics online teaching practices be improved and to validate the themes proposed in this study. The attendees of the meeting served as the external validators. The external validators involved two mathematics teachers (both female with at least 25 years of teaching experience), one LMS administrator (female with 20 years of work experience; conducts LMS training and develops University-wide online course materials), and one academic administrator. All external validators agreed with the themes and recommendations of the study.

## Results

### RQ1: Learners-related factors

It is found that 98% of the online learners own 1 or 2 devices (see Table 1). More than half of the respondents do not have personal learning space during online learning sessions. Seventy percent reported that intermittent Internet connection is the most problematic aspect of online learning. They perceived that mathematics delivered in an online platform is harder to learn than in a face-to-face setup. A large percentage of online learners (89%) mainly rely on lectures from teachers or from the help of their classmates to understand mathematical concepts. Fifty-six percent of the students rely on their teacher’s or classmates’ consultation to understand the lessons.

**Table 1:** Learners-related factors

Learners-related factors	Frequency	Percentage
Device ownership		
One device	17	31
Two devices	36	67
Three devices	1	2
Personal space for online learning		
With personal space	28	52
Without personal space	26	48
Internet connection		
Very slow	3	6
Slow	5	9
Sometimes fast, sometimes slow	38	70
Fast	7	13
Very fast	1	2
Perception of mathematics online learning		
Harder than face-to-face	34	63
Easier than face-to-face	3	6
About the same level of difficulty with face-to-face	5	9
About the same level of ease with face-to-face	0	0
Unsure/I cannot tell	12	22
Mathematics learning autonomy		
I can understand the topics by just reading the materials all by myself	1	2
Most of the time, I can understand the lesson by myself. I seldom consult my teachers or my classmates	5	9
Sometimes I understand the lesson; sometimes I do not understand it. I consult my teachers or my friends for further explanations	30	56
Most of the time, I just rely on the lectures of my teachers or tutorials of my classmates and friends	11	20
I entirely rely on my teachers’ lectures or from the help of my classmates and friends	7	13

### RQ2: Mathematics self-concept in an online learning setup

**Fig. 1:**
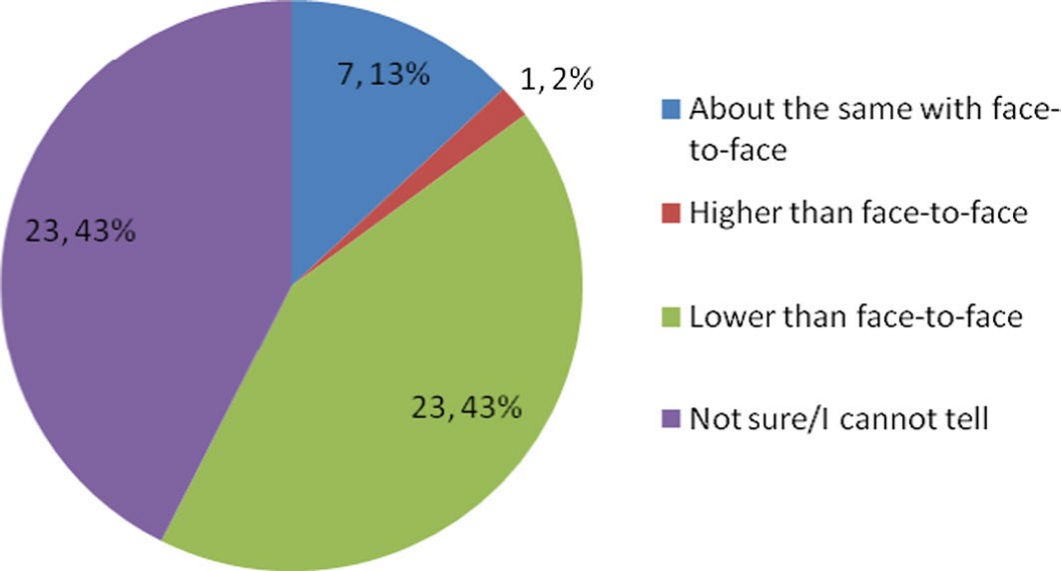
Perceived mathematics performance in an online learning setup

Online learners have negative notions about their capabilities in terms of understanding the lessons, solving problems easily, finishing the course, performing better relative to their classmates’ abilities, and performing better relative to their schoolmates’ abilities (Table 2). They feel it is more enjoyable to learn in a face-to-face setting than online. In terms of mathematics performance, 43% of the students feel that they will get lower grades in the online course than when it is done in a face-to-face session. Another 43% reported that they are unsure of what grade they will get at the end of the semester (Fig. 1). This perceived academic performance could be attributed to the unfamiliarity of the new learning environment. Students feel unprepared for online learning (Daniel, 2020; Fabito et al., 2021; Murphy, 2020) and they may feel it undesirable (Aguilera-Hermida, 2020).

**Table 2:** Mathematics self-concept in terms of ability and interest

Mathematics self-concept in online setting	*M*	SD
Ability		
I can get good grades in the course. (Good Grades)	0.11	0.66
I can understand the lessons easily. (Understand Lesson)	− 0.17	0.75
I can solve problems easily. (Solve Problem)	− 0.19	0.73
I can do well overall in the course. (Do Well)	0.15	0.71
I can attend the class easily. (Attend Class)	0.94	0.96
I can do the assignments easily. (Do Assignments)	0.46	0.79
I can help my classmates with our assignments. (Help Classmates)	0.31	0.75
I can easily recall what I have learned. (Recall)	0.07	0.77
I can finish the course online than face-to-face. (Finish Course)	− 0.26	0.87
I can perform better than my classmates. (Better than Classmates)	− 0.43	0.82
I can perform better than my schoolmates. (Better than Schoolmates)	− 0.37	0.73
I can pass the course. (Pass Course)	0.91	0.83
Interest		
I will be more interested to learn. (Interested to Learn)	0.43	1.04
I will enjoy more the lecture online than face-to-face. (Enjoy Lecture)	− 0.52	1.06
Overall Mean	0.10	0.52

Despite these negative notions, students have positive outlooks in terms of achieving good grades, doing well in the course, attending classes, doing assignments, helping their classmates in their assignments, recalling the lessons, and passing the course. They perceive that learning through the LMS is interesting. These findings suggest that they believe that their abilities can still meet the demands of the course. They are confident that they can still perform well despite the challenges and uncertainties they are facing.

### RQ3: Relationship between learners-related factors and mathematics self-concept

It was also determined the relationship between the profile of online learners and their mathematics self-concept (Table 3). The number of devices they can use has positive relationship with understanding (*r* = 0.27, *p* < 0.05) and recalling (*r* = 0.34, *p* < 0.05) the lesson. Perceived Internet speed is positively related to the ease of attending class (*r* = 0.29, *p* < 0.05). Statistical analyses found that correlations exist between mathematics learning autonomy and mathematics self-concept. Out of the 15 correlation analyses, six variables have significant correlations with mathematics learning autonomy. Mathematics learning autonomy have positive relationship with the abilities to get good grades (*r* = 0.42), solve problems easily (*r* = 0.40), do assignments easily (*r* = 0.31), recall the lessons (*r* = 0.34), and perform better than their classmates (*r* = 0.40) or schoolmates (*r* = 0.39).

**Table 3:** Spearman rank correlation between online learners’ profile and mathematics self-concept

Mathematics self-concept in online setting	Number of devices	Perceived internet speed	Math learning autonomy
Ability			
Good grades	*r* = 0.19; *p* = 0.15	*r* = 0.24; *p* = 0.08	*r* = 0.42; *p* = 0.002
Understand lesson	*r* = 0.27; *p* = 0.047	*r* = 0.18; *p* = 0.18	*r* = 0.18; *p* = 0.18
Solve problem	*r* = 0.18; *p* = 0.21	*r* = 0.18; *p* = 0.20	*r* = 0.40; *p* = 0.003
Do well	*r* = 0.21; *p* = 0.12	*r* = 0.22; *p* = 0.12	*r* = 0.31; *p* = 0.024
Attend class	*r* = 0.07; *p* = 0.64	*r* = 0.29; *p* = 0.034	*r* = 0.19; *p* = 0.17
Do assignments	*r* = 0.23; *p* = 0.09	*r* = -0.04; *p* = 0.791	*r* = 0.31; *p* = 0.024
Help classmates	*r* = 0.23; *p* = 0.09	*r* = -0.04; *p* = 0.79	*r* = 0.31; *p* = 0.024
Recall	*r* = 0.34; *p* = 0.013	*r* = 0.13; *p* = 0.335	*r* = 0.34; *p* = 0.011
Finish course	*r* = 0.18; *p* = 0.20	*r* = 0.25; *p* = 0.064	*r* = 0.17; *p* = 0.22
Better than classmates	*r* = 0.21; *p* = 0.13	*r* = 0.15; *p* = 0.28	*r* = 0.40; *p* = 0.003
Better than schoolmates	*r* = 0.17; *p* = 0.21	*r* = 0.08; *p* = 0.57	*r* = 0.39; *p* = 0.004
Pass course	*r* = 0.094; *p* = 0.49	*r* = 0.09; *p* = 0.50	*r* = 0.14; *p* = 0.33
Interest			
Interested to learn	*r* = 0.21; *p* = 0.13	*r* = 0.03; *p* = 0.82	*r* = -0.01; *p* = 0.95
Enjoy lecture	*r* = 0.03; *p* = 0.82	*r* = − 0.05; *p* = 0.61	*r* = − 0.05; *p* = 0.70
Perceived mathematics grade	*r* = − 0.18; *p* = 0.18	*r* = − 0.26; *p* = 0.60	*r* = − 0.16, *p* = 0.26

### RQ4: Difference in the mathematics self-concept of online learners between with and without personal learning spaces

Further analysis was conducted to determine if mathematics self-concept differs between online learners with and without personal learning spaces. Both online learners (with personal learning space, 30%; without learning space, 33%) agreed that mathematics learning in an online environment is harder than face-to-face. However, they have different opinions in terms of their mathematics grades. Online learners with no learning space perceived that they might have a lower grade than in a face-to-face course (30%) while those who have personal learning spaces are unsure of what grades they will get at the end of the semester (28%). The test of the difference between the means of self-concept of learners explains these results.

Mann Whitney *U* test revealed that the mathematics self-concept of the two groups of learners differ significantly in terms of achieving good grades (*U*(52) = 252.0), solving problems easily (*U*(52) = 243.5), doing well in the course (*U*(52) = 249.5), answering assignments (*U*(52) = 256.0), and recalling the lessons (*U*(52) = 225.0) (Table 4). The results are unlikely to have arisen from sampling error (*p* < 0.05). Online learners with no personal learning space had lower mathematics concepts than privileged online learners.

**Table 4:** Mann–Whitney U test between math self-concept of online learners with and without access to personal learning space

Math self-concept	*Mean Rank* (Without access, n = 28)	*Mean Rank* (With access, n = 26)	*U* (df = 52)	*p *value
Ability				
Good grades	23.5	31.8	252.0	0.020
Understand lesson	24.5	30.7	280.0	0.09
Solve problem	23.2	32.1	243.5	0.012
Do well	23.4	31.9	249.5	0.024
Attend class	25.4	29.8	305.0	0.274
Do assignments	23.6	31.6	256.0	0.042
Help classmates	25.0	30.2	294.5	0.177
Recall	22.5	32.8	225.0	0.009
Finish course	26.7	28.4	342.0	0.678
Better than classmates	23.9	31.4	264.0	0.055
Better than schoolmates	24.5	30.7	280.0	0.104
Pass course	24.8	30.4	287.5	0.161
Interest				
Interested to learn	26.9	28.2	346.5	0.752
Enjoy lecture	29.3	25.6	314.5	0.368
Perceived mathematics grade	30.7	24.0	274.0	0.09

### RQ5: Challenges on online learning

Table 5 shows the challenges that the informants faced in learning mathematics in an online environment. All validators agreed that they experienced these challenges. All the informants and validators alike agreed that technological challenges are the most pressing concern in online learning. Only one of the informants reported power interruption. This informant is in the province and his province has been experiencing regular power interruptions. This statement confirms the study of Bhuasiri et al. (2012). The other informants and validators may not experience this because they are all in the National Capital Region where power interruption is rare.

**Table 5:** Challenges faced by the respondents in mathematics online learning

Challenges	Definitions
Technological • Intermittent Internet connection • Power interruption	It refers to the essential technologies to support online learning
Personal • Lack of focus • Procrastination • Less productivity	It refers to the difficulties that students have to deal with personally
Domestic • Running for errands • Household chores responsibilities • Uncontrolled situations • Noise and other distractions	It refers to the distractions at home
Assessment • Few practice drills • Examination design • Inadequate time allotment to answer problems • Clarity of instructions • Familiarity with the system • Assessment feedback • Submission deadlines	It refers to the design of the strategies of the teacher to evaluate the mathematics skills of the students
Pedagogical • Understanding the topics	It refers to the difficulty of learning the topics because of the inappropriateness of content delivery
Consultation • Difficulty to seek help from friends or classmates • Difficulty to seek help from teachers	It refers to the difficulty of seeking clarifications from teachers, classmates, and friends
Test Anxiety • The anxiety of taking the exams online	It refers to the feeling of being nervous when taking online quizzes or examinations

The second challenge involves a problem that can only be solved by the students themselves. One of the informants said:

It is hard to focus on my studies. Unlike in a classroom setup, the environment is conducive to learning. All you need to do is to listen to the teacher. When you are at school, your mind is conditioned to study. When you are at home, you are in a comfort zone. I tend to do other things and delay doing my assignments. I admit: I become less productive and do not manage my time well when I am at home.

However, it must be noted that one of the validators did not agree that he procrastinates. This is the only item that the validators disagree with. Domestic challenges contribute to the distraction of students' online learning. Even while during class sessions, students were asked to run for errands or do simple household chores. Some situations are beyond the control of the students and their families (e.g., visitors). Noise and distractions contribute to domestic challenges. This finding confirms the study of Baticulon et al. (2021) and Fabito et al. (2021). One informant commented:

I have other responsibilities at home. Sometimes, I feel guilty because they are all busy doing household chores while I am on just on my computer throughout the day. Sometimes, I have to run errands. There are times that I have to respond to our neighbors' calls who are looking for my parents.

One validator agreed and said:

I agree with this. I want to also add that I only used the living room for my online learning sessions. Sometimes, the people in the house forgot that I am having an online learning session. They play music while walking around the living room. There was even an incident that they look at my laptop thinking that I was only watching movies.

Informants and validators reported that they experienced assessment challenges. Assessment challenges involve few practice drills, design of the online examination, clarity of instructions, and assessment feedback. One informant commented that teachers only provide about three questions and let them study and solve the other problems. This practice is construed as ineffective because, according to the informant, practice is an important activity that builds up their mathematics skills. The informant explained that this could be attributed to the desire of the teacher to cover the whole content of the course syllabus. When he was asked whether he prefers quality over quantity of the content, he chose the former. The validator stated: “There is no point in covering the whole syllabus when you did not understand any of them.”

The design of the examination refers to the way the questions are presented on an online platform. These include questions that require answers with long inputs of formula that are susceptible to typographical errors, multiple types of questions that are prone to guessing, time allotment, unclear instructions, and familiarity with the system itself. Informants and validators pointed out those problems that need to input their solutions in a text box entail a lot of time. They explained that they solve the problems on a piece of paper and then transfer them to the online submission system. They also raised their concerns on submission deadlines as they also have other courses with the same course requirements to satisfy. They also requested feedback on their activities and quiz results so that they would not commit the same mistakes.

The informants and validators further pointed out that they need more time as they familiarized themselves using the system. This is consistent with the study of Binti Abd Aziz et al. (2020). One informant said: “In an online exam, you are not only concerned with the correctness of your answers. You are also concerned about how you will input your answers correctly in the system. Teachers have to take into account that we also need time to familiarize ourselves with the system.”

The informants and validators also raised a pedagogical issue. The study of Baticulon et al. (2021) classified understanding the content of the course as a personal barrier. However, this is not the case based on the interviews with the informants and validators. According to informants and validators, it is difficult to understand the topics in online learning because of its delivery. When it is delivered appropriately, they can understand the topics and have better chances of passing the quizzes and exams. They prefer a combination of content delivery strategies including discussion of PowerPoint slides with step-by-step solutions through online meeting apps (e.g., Google Meet), and recorded videos of step-by-step solutions. One of the informants emphasized this comment: “Please do not let us study mathematics on our own. You do not just give the materials to us and let us understand the content.”

Before online learning, informants and validators seek the assistance of their teachers, classmates, or friends. The informants and validators understand that it is difficult to seek consultation because it is difficult to find a common time for consultation. Teachers and students have other responsibilities to attend to after online classroom sessions.

Students developed test anxiety because of the aforementioned challenges. According to the statement of one informant,

There is always a nervous factor when taking the quizzes or examinations since these are time-based activities. I am anxious since my Internet connection could suddenly become unstable or there might be a power interruption. Some teachers do not allow returning to the questions. Once you skipped the question, it will be given a zero mark. Unlike in a paper-based test, you can skip the questions and go back working on it if there is still time. I understand my teachers. They are thinking that a time-limited quiz/exam is a way to deter cheating.

## Discussion

This study investigated the classroom experience of online learners in a mathematics class during the summer of 2020. Toward this goal, the study attempted to determine the relationship between the online learners-related factors and their mathematics self-concept. Moreover, interviews were conducted to determine the challenges they faced in learning mathematics delivered on an online platform. The online-related factors in terms of device ownership revealed that they own 1 or 2 devices. Access to the device is not a problem to this set of participants relative to the general student population that may experience the digital divide (Cavanaugh et al., 2009; Pena-Bandalaria, 2009). This can be explained by the fact that the participants of this study are IT students, where learning activities, even before the pandemic, are highly dependent on devices.

The quantitative result shows that Internet connection is the most problematic aspect of online learning. An intermittent Internet connection can greatly affect the attendance of the students in online classes. This finding is consistent with the interview results in terms of technological challenges. This is a national problem since the Philippines has slow Internet connectivity (Chiu et al., 2017). According to Natividad (2021), and Salac and Kim (2016), the Philippines has a slow Internet connection because of the outdated Philippine law and red tape that hinders the quick installations of cell towers. This result confirms the findings of Bhuasiri et al. (2012) and Baticulon et al. (2021). Although only one of the informants reported an issue of power interruption, his concern is valid. His concern might not be similar to other informants or validators simply because the other informants and validators are all living in Metro Manila.

Almost half of the participants have no personal learning space during online learning sessions. Online learners with no personal learning space had lower mathematics concepts than privileged online learners. The lack of personal learning space during online sessions puts online learners in a disadvantaged position to attain an optimal learning experience. This is consistent with the findings of Baticulon et al. (2021), and Fabito et al. (2021). As explained in the interviews, students who lack personal learning space are more susceptible to distractions during, and even after online learning sessions. Noise and running errands are the most common forms of distraction. The interview results show that other members of the family may simply forget the students are in an online class. In short, as one student commented, access to personal physical learning space can create an environment conducive to online learning.

Online learners disclosed that they understood the content through lectures and constant consultation with teachers. This is consistent with the interview results that students dislike studying the course content on their own. This mathematics learning autonomy is the exact opposite of the nature of asynchronous learning. In asynchronous learning sessions, students have to study a lecture on their own. In other words, learners who are consultation-dependent will resist this educational shift.

As shown in Table 3, students with low learning autonomy are expected to have lower dispositions of their mathematics abilities. Students who feel inferior about their mathematical abilities tend to have lower mathematics performance (Lee & Kung, 2018). In students’ point of view, asynchronous session activities (e.g., reading materials, assignments, practice drills, and quizzes) are challenging in the aspects of assessment, pedagogy, and consultation. These challenges explain why students experience test anxiety. Consequently, these difficulties contributed to their feeling of uncertain or low grade perceptions. The quantitative results provide insights to address this issue, i.e., students have to be gradually introduced into the concept of learners’ autonomy. Furthermore, family members may dedicate a place in the house that will serve as an online learning space.

The shift to an educational setting had a negative impact on the mathematics self-concept of learners. More than 80% of the respondents perceived that they will have a lower grade in mathematics. They also have negative notions of their mathematics self-concept in terms of understanding the lesson, solving problems, finishing the course, performing better relative to their classmates or schoolmates, and enjoying the online class. These negative notions on their capabilities and interest in online learning can be explained by the fact that full online learning is just implemented recently. While online learners have experience using the LMS before the COVID-19 pandemic, they are not yet fully familiar with a fully online learning setup. This is evident in one of the narratives of the informants. On one hand, the positive mathematics self-concept indicates that they are hopeful in the aspects of achieving good grades, attending classes, doing assignments, helping their classmates in their assignments, recalling lectures, passing the course, having the interest to learn, and doing well overall in the course. Teachers have to sustain these positive outlooks to achieve the course outcomes.

Device ownership has a positive relationship with understanding and recalling the lecture. Multiple devices such as laptops and mobile devices are dependable for students' online learning (Muyinda et al., 2010). Multiple device ownership allows online learners to view multiple screens and to store multiple copies of learning materials (Pynos, 2016). Multiple device usage in learning also creates seamless connectivity that enables the continuity of the learning experience (Milrad et al., 2013). This practice allows easy access to information that is useful for solving problems. For example, an online learner may be looking at his/her laptop screen for the given problem while he/she is looking into another device (e.g., cellphone) that displays the formula and the sample solved problems. Furthermore, multiple devices can address accessibility or installation issues.

Perceived Internet speed is positively related to the ease of attending class. This finding is expected. What is more interesting is that perceived Internet speed does not relate to the other items of mathematics self-concept. The results imply that a fast Internet connection is only necessary to attend the class but not necessarily related to the online learners' perceptions about their mathematics abilities. Their perceptions about their abilities and interest in mathematics are not related to the speed of Internet access. In other words, there is no link between the confidence of online learners in their mathematics abilities and their speed of Internet access.

Meanwhile, mathematics learning autonomy is correlated with most of the mathematics self-concept. This vivid finding denotes that mathematics self-concept is mostly related to the perceived abilities of online learners to study at their own pace, i.e., as students become more independent learners, they tend to have a higher mathematics self-concept. Teachers have to emphasize to the online learners that online learning is different from face-to-face where teachers can intervene when confusion or challenges arise in understanding the lessons. Teachers, at the onset of the course, are encouraged to orient online learners that they are expected to be independent learners. Problem sets and learning materials may be given in advance to develop the habit of independent learning.

Consistent with the literature, the respondents of this study experienced technological, personal, and domestic challenges. There is a challenge that students can be addressed by themselves (e.g., procrastination) but most are beyond their control. Domestic challenges require the support and understanding of family members. The students and their family members must have open communication. They should set house rules in terms of household chores and running errands.

The study of Baticulon et al. (2021) categorized the inability to understand the content of the course as a personal barrier to online learning. In this study, it was shown that this is a pedagogical challenge than a personal problem. Furthermore, it was disclosed that teachers have direct responsibilities on four out of the seven identified challenges. Challenges in the teaching and assessment had the most number of concerns. These results guide teachers to devise creative teaching and fair assessment strategies that could address these concerns. For teaching and learning activities, teachers are advised to provide ample time for lectures and deliver the contents through different forms of multimedia. At the end of each lecture, teachers may elicit feedback from students to assess if the students understood the lessons. It is advisable to gather feedback from struggling as well as high-performing students to understand the challenges of the students with diverse mathematical abilities. Group learning activities may be conducted using the Group Discussion function of the LMS. Teachers may provide practice drills that are not yet included for grade computation. Provide 2–3 days to allow students to do their assignments. The asynchronous sessions may also be utilized as consultation time. Teachers may use the randomized function of the LMS to pick random questions from its databank. Teachers may also request students to show their computer windows during the quiz (see “Appendix A”).

Another important role of the teachers is to sustain the positive and counter the negative mathematics self-concept of the students. Teachers at the onset of the course, the questionnaire here may be utilized to determine the mathematics self-concept of the students. Students should be oriented about the course expectations. Teachers should introduce independent learning gradually (“Appendix A”).

## Conclusions, recommendations, limitations, and implications

This study investigated the profile of online learners and its influence on their mathematics self-concept. It is revealed that online learners in this study have access to devices. Physical learning space is one important aspect of an online learning environment. However, some online learners have physical learning space limitations which make online learning inconvenient. This limitation contributed to their low academic self-concept.

The majority had reported an intermittent Internet connection. Online learners have mixed notions about their mathematics capabilities and interest in learning mathematics in an online environment. They expressed uncertainties about the possible grades they will get at the end of the semester. The ability of online learners to study mathematics at their own pace is the most desired skill for online learners. Moreover, online learners with limited learning space are more likely to experience a lower mathematics self-concept because they cannot focus on the course. Thus, it can be concluded that the profile of online learners partly influences their mathematics self-concept.

Teachers play a significant role in improving and sustaining the mathematics self-concept of online learners. At the beginning of the class, teachers must inform online learners that having a habit of self-paced learning is a highly desirable discipline. Teachers have to sustain the positive mathematics self-concepts of online learners. They may assure online learners that online consultations are available when needed. Timely feedback on the works of online learners is highly encouraged to sustain their positive outlook about their capabilities. Individualized feedback can be provided to inform online learners that they are performing well (or not performing well) relative to his/her classmates.

The negative mathematics self-concepts of online learners serve as a basis for teachers to find ways to address these negative notions. Teachers have to be creative in delivering the content of the course (O’Doherty et al., 2018). For instance, PowerPoint slides with a voice recording or a previous video recording of the lesson may be utilized for lecture sessions. These materials may be accessed anytime and students with a slow Internet connection can still follow the phase of the course. Teachers may conduct synchronous learning sessions to answer questions or clarifications. An unwavering teacher’s dedication and understanding are suggested to assist online learners to finish the course.

The study is limited in terms of the participants and sample size. These limitations existed because of the timing of the shift of mode instructions in the university. There were only limited courses offered and a small number of students were enrolled when the study was conducted. Thus, the findings of the study may not be widely applicable beyond this population. Despite these limitations, this study provides clear insights into the students' mathematics self-concept and the challenges they faced in an online learning environment. The realities discovered in this study cannot be denied and deserves the attention of mathematics teachers. Nevertheless, a university-wide investigation of mathematics self-concept may be initiated to improve further the findings of the study.

There are issues raised in the study that cannot be solved by teachers. The members of the family must understand that online learners need physical learning space and minimal disruptions. To address this concern, teachers, or schools may send letters to parents about online learning to observe the online learning schedule of their children. Cooperation and understanding from family members are necessary for providing an environment conducive to online learning. It is strongly recommended that family members dedicate a physical learning space for online learners.

Educational institutions have to select an LMS that can support the demands of the course. The institution needs to understand the online learning requirements of the different degree programs. It is imperative to understand the strengths and limitations of the different LMS. A selection criteria committee may be instituted to select an LMS and to review its effectiveness relative to the needs of the students and faculty. Usability testing of the LMS may be done after the implementation. This will identify the ease of use and satisfaction of use of the LMS. The evaluation process may also evaluate whether the LMS supported the pedagogical requirements of the faculty (Pipan et al., 2008).

Another challenge the institution facing is the possibility of students that might be left behind because of inadequate access to devices. The formation of a technical support group is also desirable. Educational institutions may extend their help to online learners by lending laptops, tablets, and mobile Wi-Fi. Local government units may also offer assistance to underprivileged students. For example, a city local government unit in the Philippines provided online learning devices (e.g., laptops or tablets) to students (Casinas, 2020) and installed Internet centers to support online learning (Kabagani, 2020).

Lastly, the government may reinforce fast Internet connections through legislation. One of the possible legislations is to shorten the application of business process applications of constructing Internet facilities (Natividad, 2021; Salac & Kim, 2016). The government may allocate funds for the development of Internet infrastructures. These funds may be directed to rural areas. A government-private partnership may also be initiated. With this partnership, lengthy bureaucratic procedures will be avoided. Finally, the government may promote a market of competitiveness through the inclusions of other Internet providers (Salac & Kim, 2016).

## Data Availability

Data cannot be shared because of existing laws in the country of the authors.

## Appendix A: Checklist of suggestions for online mathematics teaching

Mathematics Self-concept in an Online Setting

- Determine the mathematics self-concept of the students at the start of the class.
- Inform the students of the setup of the course.
- Introduce learners to the concept of independent learning.
- Assist students in re-enforcing positive self-concepts.

Teaching Learning Activities

- Provide ample time for lectures.
- Immediately elicit feedback after the lecture.
- Gather feedback from high-performing and struggling students about the phase and clarity of the lecture.
- Use the combination of PowerPoint slides, online meetings, and videos for course content delivery.
- Make the due dates reasonable (2–3 days).
- Use the Group Discussion function of the LMS to encourage group study among students.

Assessment and Consultation

- Provide individualized feedback on students’ activities.
- Inform students about their class performance.
- Dedicate synchronous sessions intended for consultation or feedback.
- Provide more seatwork and practice drills.
- Provide assessment activities that are not recorded.
- Balance the types of the assessment – e.g., minimize multiple-choice, more on problems showing solutions, and discourage providing answers that are format-sensitive.
- Balance the difficulty levels of the assessment.
- Use the randomized function of the LMS to generate quizzes and exam questions.

## Abbreviations

ASC: Academic self-concept
COVID-19: Coronavirus disease
IT: Information technology
LMS: Learning management system
SRL: Self-regulated learning
TMI: Traditional mathematics instructions
U: Mann–Whitney U test
WBMI: Web-based mathematics instructions
